# Primary Cutaneous B-Cell Lymphoma Co-Existing with Mycosis Fungoides—A Case Report and Overview of the Literature

**DOI:** 10.3390/life12122067

**Published:** 2022-12-09

**Authors:** Doriana Sorina Chilom, Simona Sorina Farcaș, Nicoleta Ioana Andreescu

**Affiliations:** 1 Department of Dermatology, University of Medicine and Pharmacy “Victor Babeş”, Eftimie Murgu Sq. no.2, 300041 Timisoara, Romania; 2 Department of Microscopic Morphology—Genetics, Center of Genomic Medicine, University of Medicine and Pharmacy “Victor Babes”, Eftimie Murgu Sq. no.2, 300041 Timisoara, Romania

**Keywords:** B-cell lymphoma, Mycosis Fungoides, composite lymphomas, primary cutaneous lymphoma

## Abstract

The existence of two sequential lymphomas, one localized and one systemic, either both with B or T lymphocytes, or one with B cells and one with T cells, with the same patient, is a known possibility. The second lymphoma is often induced by immunodepression or by the initial treatment. However, the existence of two cutaneous lymphomas with different cell lines, without systemic involvement, represents an uncommon situation. In this report, we describe the case of a 37-year-old man with an initial diagnosis of PMZBCL that over 10 months also developed a MF patch/plaque on the left leg.

## 1. Introduction

Primary cutaneous lymphomas (PCLs) are a group of T- and B-cell lymphomas that affect the cutaneous tissue without involving other organs at the time of diagnosis. PCLs are defined as non-Hodgkin lymphomas presenting in the skin with no evidence of extracutaneous disease at the time of diagnosis. The prevalence of PCL is of around 0.90/100,000 inhabitants in Europe and the USA [1]. Out of the total number of PCL, those with T-lymphocytes are the most common. In Western Europe, Primary cutaneous T-cell lymphomas (PCTCLs) represent about 75–80% of PCLs, while B-cell lymphomas (CBCLs) represent only 20–25% [2]. 

The most common type of PCTCLs is Mycosis Fungoides (MF), about 39% of PCTCL [2], and represents a proliferation of small to medium T-lymphocytes with hyper convoluted cerebriform nuclei. The risk factors for MF are male sex, advanced age and black race. MF develops from peripheral epidermotropic lymphocytes, which immunophenotype is positive for CD2, CD3, CD4 and CD5 and negative for CD8 and CD7 [3]. 

Primary marginal zone b-cell lymphoma (PMZBCL) is a rare disease, included in the group of extranodal marginal zone lymphoma of mucosa-associated lymphoid tissue (MALT). It is a low-grade malignant B-cell lymphoma that disseminates exceptionally and has a 5-year survival rate of 95% [4,5]. Immunohistochemically, the marginal zone cellular population is positive for CD20 and Bcl2, while the reactive germinal centers are positive for Bcl6, CD10 and negative for Bcl2 [6].

PCLs are extranodal non-Hodgkin lymphomas with broad clinical, histological, phenotypic, genetic, and prognostic spectrums. Therefore, it is necessary to collaborate in a large team of dermatologists, hematologists, pathologists, oncologists, radiotherapists and other specialists in order to obtain an accurate diagnosis and a targeted and personalized treatment. Even though CTCLs are a common lymphoproliferation in malignant skin pathology, the existence of CTCLs and CBCLs at the same time is a rare situation, with few cases being cited. 

The coexistence of two lymphomas at a single anatomical site, is defined as composite lymphomas (CL). Immunohistochemical and genetic analyses have revealed that some types of lymphomas, classified as CL, represent the morphological manifestation of the same neoplastic clones. So, the presence of two lymphomas with different neoplastic cells, could represent a coexistence of them, without a proven association between the two lymphomas [7]. 

The aim of this study is to present a rare case of PMZBCL coexisting with MF plaque stage on a 37-year-old patient. The diagnosis of the two cutaneous lymphomas was based on the histopathological and immunohistochemical examination, and their primary character was established by excluding the involvement of other organs, except the skin.

## 2. Case Report

**Figure 1:**
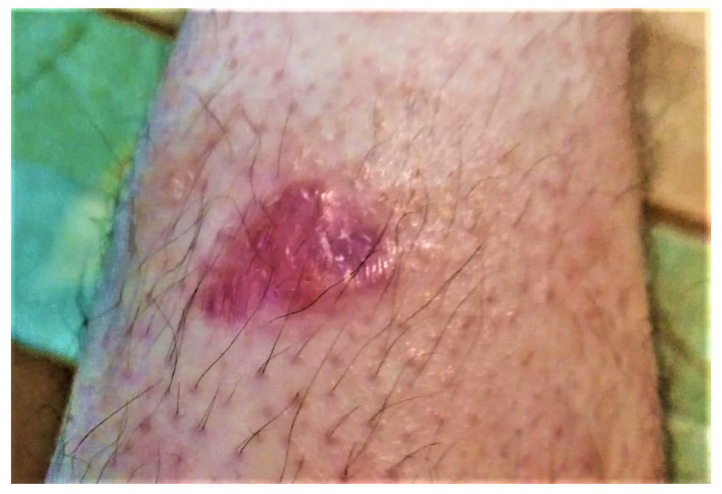
Primary marginal zone B-cell Lymphoma on the antero-lateral part of the lower left leg; purple–red plaque with shiny surface.

A 37-year-old man was evaluated at the University Dermatology Department of the Timisoara Municipal Emergency Hospital for a 5-year history of a non-itchy, papulo-erythematous plaque of 2–3.5 cm with a shiny surface on the antero-lateral part of the lower left leg, thus suggesting a granulomatous structure (Figure 1). The lesion appeared after mild, repeated traumas and began to grow in size in recent months. At the general clinical examination, the patient had a good general condition without palpable superficial adenopathy. Complete blood count and blood biochemistry (Serum creatinine, Alkaline phosphatase, GT Gamma, Blood glucose, TGO/AST, TGP/ALT, Serum urea) showed normal values, abdominal ultrasound and chest X-ray showed no pathological changes and the Mantoux test was negative. Following the first examination, the suspicion of cutaneous sarcoidosis with non-specific lesions was raised and treatment with topical corticoid of medium potency was initiated. There was no improvement after two weeks of topical treatment and so it was decided to perform a biopsy incision with histopathological examination. 

**Figure 2:**
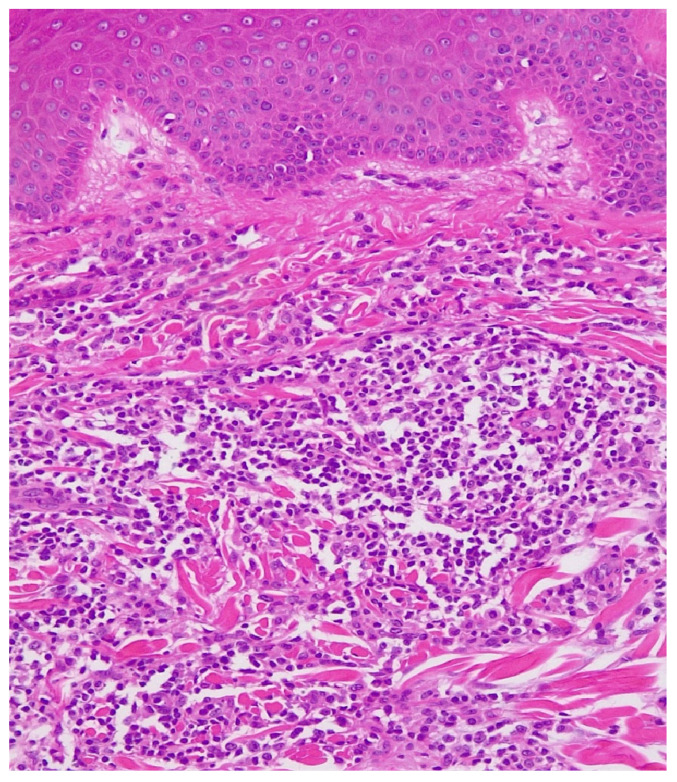
Primary marginal zone B-cell Lymphoma, Hematoxylin & Eosin stain, Ob ×20.

Histopathological (Figure 2) examination revealed ortokeratinized epidermis, hyalinization of the dermo-epidermal junction and flattening of the epidermal ridges. Papillary and deep dermis presented lymphoid proliferation with follicular and diffuse pattern that dissected the fascia of collagen fibers with periadnexial and perifollicular disposition. Lymphoid proliferation is composed of lymphoblasts, lymphocytes of centrocyte type-like with irregular nucleus and dense chromatin, quantitatively reduced cytoplasm, small lymphocytes, rare plasma cells, rare immunoblasts and very rare eosinophilic granulocytes. In different areas, the subcutaneous adipose tissue presented lymphoid infiltrates.

**Figure 3:**
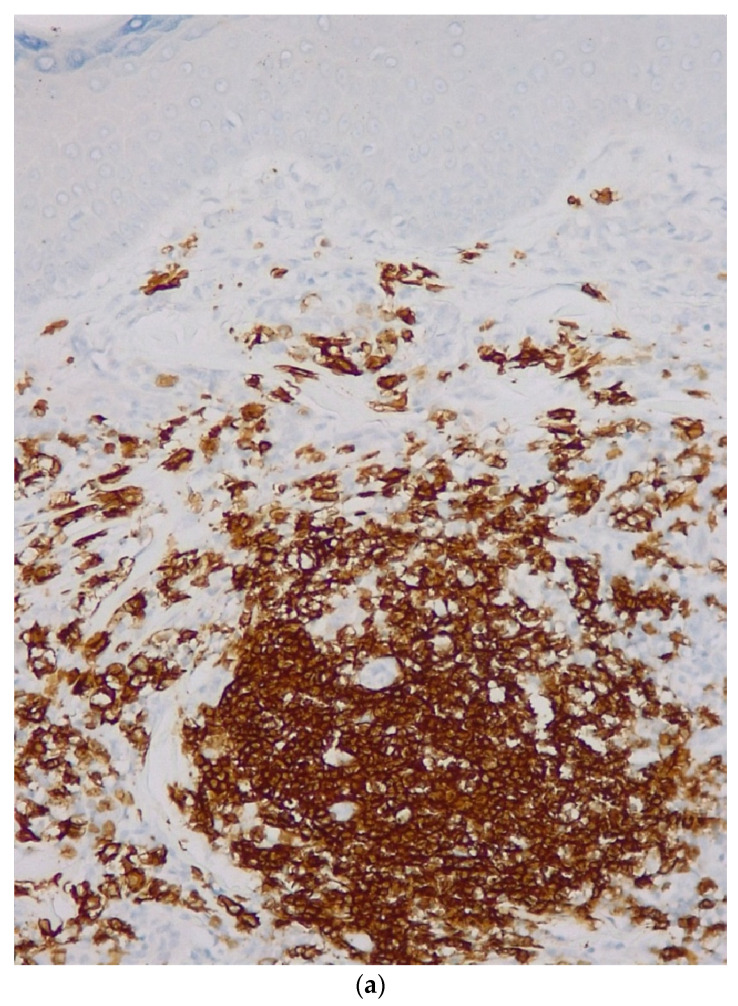

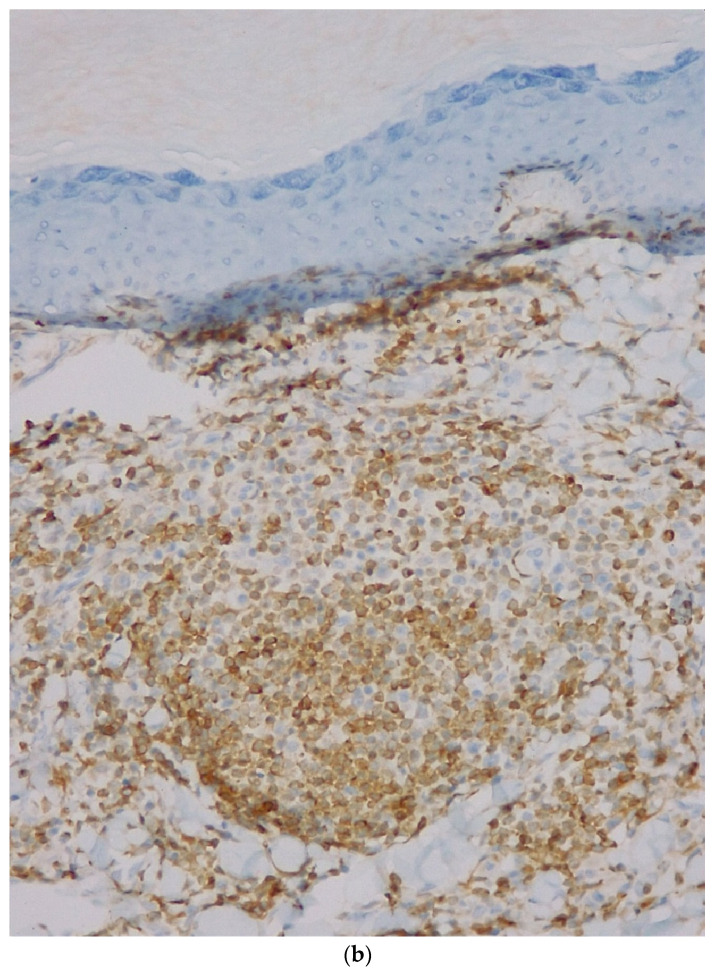
(**a**): Primary marginal zone B-cell Lymphoma, CD20 Ob ×20. (**b**): Primary marginal zone B-cell Lymphoma, Bcl-2, Ob ×20.

Immunohistochemistry (Table 1) revealed CD20-intense membrane immunoreaction in B-lymphocytes with nodular and focal interfollicular organization; Bcl2—intense cytoplasmic immunoreaction in B-lymphocytes in follicular and interfollicular areas, plasmocytes have clonal character (kappa/lambda ratio >10/1), positive for CD138, negative tumor proliferation for Bcl6 (positive in the remnants of reactive germination centers), negative for CD3 and the proliferation index measured by Ki67 was 10%. Based on these findings the diagnosis was B-cell lymphoma of the marginal area with immunophenotype CD20 positive (Figure 3a), Bcl2 positive (Figure 3b), and Ki67 = 10%.

To confirm the primitive cutaneous character of the proliferation, interdisciplinary consults and additional investigations were performed. The CT for abdomen-thorax-pelvis using contrast substance showed no thoraco-abdomino-pelvic secondary determinations and no adenopathy in the examined segments. Gastroscopy and colonoscopy presented a normal appearance; Osteo-medullary biopsy and aspirated medullary revealed medullary lymphocytes within normal limits and IgG and IgM for Borrelia were negative. Diagnosis of primary cutaneous lymphoma with B cells of the marginal area was established according to the data. 

The patient received curative local radiotherapy using a Total dose = 40 gray/20 fractions (TD = 40Gy/20 fr). The treatment was well-tolerated.

**Figure 4:**
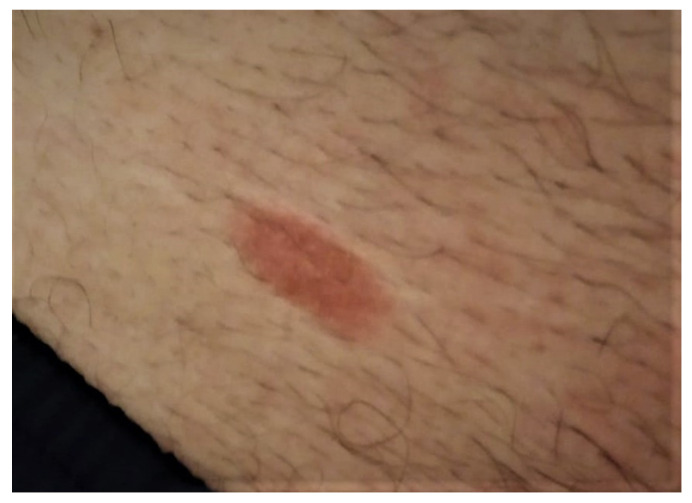
Mycosis Fungoides plaque at the right inguinal fold; oval erythematous and indurated plaque, with a fine scales surface.

After ten months from the initial diagnosis of primary cutaneous B-cell lymphoma of the marginal area, the patient returned to the dermatology clinic due to the appearance of a new plaque on the right inguinal fold (Figure 4). The plaque had an erythematous-squamous appearance, with slightly indurated well-defined edges and absent local itching or pain. The onset was reported about two months after minor trauma. The lesion has a tendency for superficial spread on the surface.

**Figure 5:**
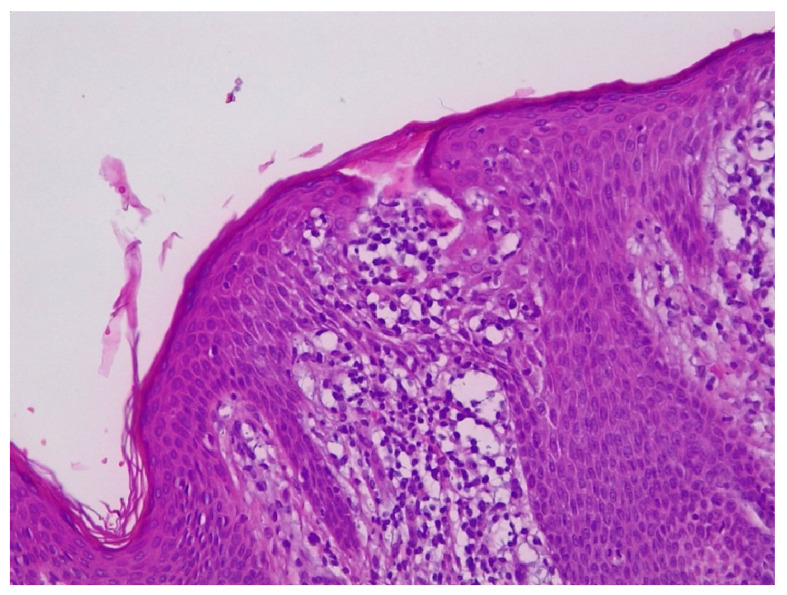
Mycosis Fungoides, Hematoxylin & Eosin stain, Ob ×20.

A histopathological examination (Figure 5) was conducted that revealed superficial infiltration with small to medium-sized atypical lymphatic cells showing epidermotropism arranged in patches with “string of pearls” distribution along the junctional zone, under a regularly maturing ortokeratotic epidermis. There was no development of Pautrier microabscesses. Cells showed irregularly shaped, partially cerebriform hyperchromatic nuclei and sparse cytoplasm. 

**Figure 6:**
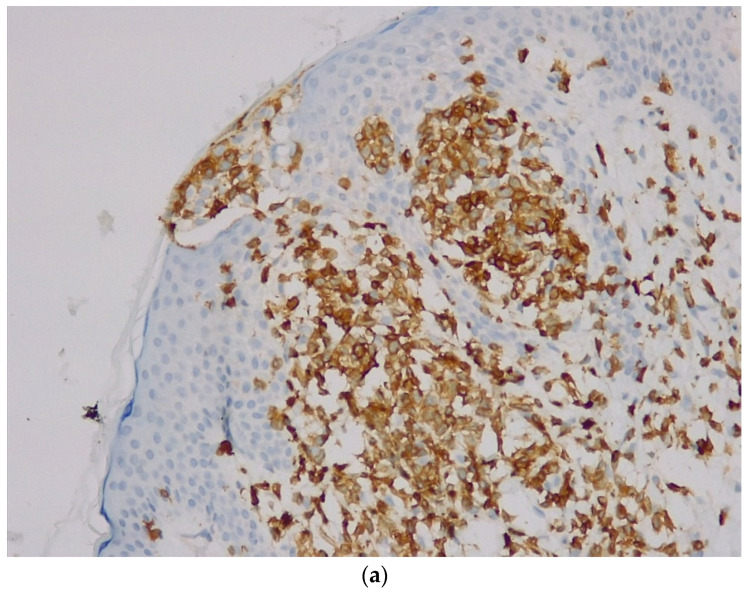

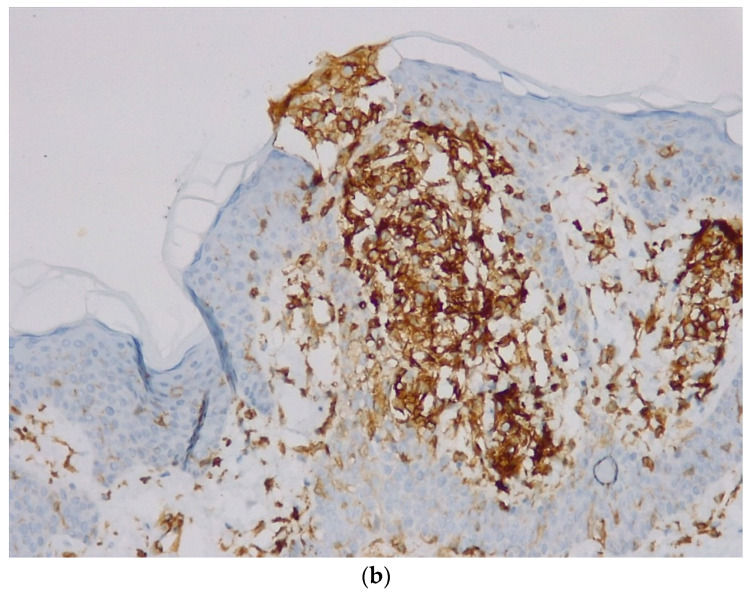
(**a**): Mycosis Fungoides, CD3, Ob ×20. (**b**): Mycosis Fungoides, CD4, Ob ×20.

The immunohistochemical test (Table 1) revealed strong CD3 (Figure 6a), CD4 (Figure 6b) and CD5 expression with almost complete CD30 negativity and complete negativity for CD8, CD56, T-cell receptor betaF1, T-cell receptor gamma-delta and a complete antigen loss for CD7. In the CD20 stain, only extremely sparse small B-lymphocytes were found. The tumor cells were negative for Bcl2 and Bcl6. The lymphoid cells were positive for CD10, and just some of them were positive for CD79 and Cyclin D1. The proliferation activity index measured by Ki67 was 20%. The edges of the excision showed free tissue. The histopathological diagnosis was early MF.

After another 3 months from the diagnosis of MF in the patch/plaque stage, the patient developed another well-defined 5 × 3 cm oval erythematous plaque located on the lateral part of the left forefoot. No other general or local symptomatology was reported. The excision of the plaque was performed with the application of a graft of healthy skin tissue taken from the inguinal area. 

Immunohistochemical examination (Table 1): infiltrated cells are consistently positive for CD20, Bcl2 with simultaneous negativity for CD5, CD10, CD23 and Bcl6, high-proliferation physiological activity (ki-67) about 10% to 20%. In CD10 and Bcl6 staining the residual partially populated germ centers emerge, which are also physiologically negative for Bcl2. The germinal centers are based on partially compact and partially fragmented networks of follicular dendritic cells (CD23). Moderately abundant CD138-positive plasma cells without light chain restriction clearly detectable in light chain staining and a continuous positivity for IgG, with simultaneous negativity for IgA, IgD and IgM are present around the nodes. In IgD and IgM staining, mantle cells surrounding partially present residual germinal centers are revealed. The results indicate dermal-epidermal tissue (of the left foot) with infiltration by indolent non-Hodgkin lymphoma of the B-cell variety—especially around the marginal zone—compatible with a primary cutaneous marginal zone lymphoma.

## 3. Treatment

The patient performed radiotherapy for PMZBCL, located on the antero-lateral part of the lower left leg (DT = 40Gy/20fr on linear ACC with 6MV. Irradiation was well tolerated). The other two lesions, MF located at the right inguinal fold and the PMZBCL located on the lateral part of the left forefoot, were excised with negative safety margins. The skin healed without local relapse. The patient is still continuously monitored in dermatological, hematological and oncological services to ensure early diagnosis of relapsing lesions.

## 4. Discussion

Composite lymphomas (CL) are a combination of either two different B-cell lymphomas, two different T-cell lymphomas, or one B-cell lymphoma and one T-cell lymphoma that develop at a single tissue site simultaneously. The development of CL could be caused by chemotherapeutic agents, immunological defects or autoimmune diseases (Sjögren’s syndrome, primary or acquired immunodeficiencies, and autoimmune lymphoproliferative syndrome) [8].

The incidence of CL is between 1% and 4.7%, and the most common associated lymphomas are two Non-Hodgkin B-cell Lymphomas, or a Non-Hodgkin B-cell lymphoma with a Hodgkin lymphoma. CL that associates T-cell lymphomas with B-cell lymphomas are rare, their location is frequently in the lymph nodes or lymphoid organs, and only rarely involve non-lymphoid organs [9].

Several mechanisms were proposed for explaining the occurrence of composite lymphomas with one being an immune deficiency secondary to an Epstein–Barr viral (EBV) infection [10]. There were several cases reported for which a EBV infection was documented, but there are also cases where the EBV infection was lacking, suggesting that other mechanisms can be involved in the development of CL [10,11,12,13]. For the case presented here, EBV infection was excluded by performing IgM and IgG serology for Epstein–Barr virus.

The appearance of a lymphoma or a new form of cancer in a patient treated for a pre-existing malignancy is a known possibility. The main cause is immunosuppression induced by the first malignancy or by its treatment. Herrmann & Sami reported a case of a 54-year-old man diagnosed with diffuse large B-cell lymphoma (DLBCL) on his right knee. A cutaneous T-cell lymphoma (CTCL MF-type) was diagnosed shortly after. The patient also had an ongoing symptomatic history of psoriasiform dermatitis starting 15 years prior, which the authors considered to represent the newly diagnosed MF. The patient received local radiation treatment for DLBCL and chemotherapy for CTCL. Shortly thereafter, he developed a tumor on the radiotherapy area that turned out to be CTLC type rather than a recurrent B-cell lymphoma [14]. In the case we are reporting, the patient received only radiotherapy, and the MF developed in a different area than the irradiated one.

A meta-analysis study which analyzed the risk for second malignancies in non-Hodgkin’s lymphoma survivors concluded that patients with non-Hodgkin’s lymphoma have a higher risk for a second malignancy than the general population even after controlling for treatment for non-Hodgkin’s lymphoma (chemotherapeutic drugs, radiotherapy, combined-modality approaches including conventional-dose chemotherapy with radiotherapy or with total body irradiation) [15]. Multiple cases of association of non-Hodgkin lymphomas with other types of lymphomas have been reported over time [16,17], but the simultaneous presence of two cutaneous lymphomas is an unusual situation. However, the appearance of the second lymphoma occurs a few years after the first diagnosis [18]. Radiation therapy can also induce a second malignancy, but this risk is not clearly quantified, and it is assumed that it occurs after a long period of time [19].

In our case, the patient developed MF ten months after the first diagnosis of B-cell lymphoma (located on the antero-lateral part of the lower left leg). It is a small possibility that MF to be caused by the radiation therapy performed to treat PMZBCL, given the short period of occurrence of the second cutaneous lymphoma. In addition, the appearance of a cutaneous lymphoma due to local radiation therapy, occurs in the area where the radiation therapy was applied. 

In regard to prognosis, most CL have an aggressive clinical course, and little data is available about treatment options and their outcomes [12]. A recent study reported a recovery rate of 60% when using a protocol including rituximab, cyclophosphamide, doxorubicin, vincristine, prednisone (R-CHOP) [20]. The patient presented here, followed the local curative radiotherapy for PMZBCL located on the antero-lateral part of the lower left leg, and the lesions of MF and the second PMZBCL, were excised within safety limits, without the need for another associated therapy. 

## 5. Conclusions

This case report presents a case of PMZBCL followed 10 months later by the appearance of MF, and another 3 months later by a new lesion of PMZBCL, which is an unusual association. The CD20, Bcl2 with simultaneous negativity for CD5, CD10, CD23 and Bcl6 is the immunohistochemical profile for PMZBCL. The cutaneous lymphoid infiltration in small/medium T cell, diffuse positive for CD3, CD4 and CD5 with simultaneous almost complete negativity for CD30 and complete negativity for CD8, CD56, T-cell receptor betaF1, T-cell receptor gamma-delta and a complete antigen loss for CD7 revealed an MF diagnosis. Analyzing these data, we consider that these two cutaneous lymphomas coexist, with no direct connection between them. This requires a detailed genetic and molecular investigations to assess the oncogenic status and to exclude overlapping diagnoses. 

## Figures and Tables

**Table 1:** Immunophenotypic features for the case presented.

Immunophenotypic Features of PMZBCL on the Antero-Lateral Antero-Lateral Part of the Lower Left Leg		Immunophenotypic Features of MF Plaque at the Right Inguinal Fold		Immunophenotypic Features of PMZBCL on the Lateral Part of the Left Forefoot	
Ki67	10%	Ki67	20%	Ki67	10–20%
CD20	positive	CD20	negative	CD20	positive
Bcl2	positive	Bcl2	negative	Bcl2	positive
CD3	negative	CD3	positive	CD3	positive
κ/λ	>10/1	CD4	positive	CD4	ND
CD5	negative	CD5	positive	CD5	negative
CD10	negative	CD7	negative	CD10	negative
CD23	negative	CD8	negative	CD23	negative
Bcl6	negative	Bcl6	negative	Bcl6	negative
CD138	positive	TCR βF1	negative	CD138	positive
		TCR γδ	negative	IgG	positive
		CD10	positive	IgA	negative
		CD56	negative	IgD	negative
		CD79	positive		
		Cyclin D1	positive		
		CD30	negative	IgM	negative

ND = not done, MF = Mycosis Fungoides, PMZBCL = Primary marginal zone B-cell Lymphoma, κ/λ = kappa/lambda ratio, TCR βF1 = T-cell receptor beta F1, TCR γδ = T-cell receptor gamma-delta.
